# Aptamers in Virology—A Consolidated Review of the Most Recent Advancements in Diagnosis and Therapy

**DOI:** 10.3390/pharmaceutics13101646

**Published:** 2021-10-09

**Authors:** Tejabhiram Yadavalli, Ipsita Volety, Deepak Shukla

**Affiliations:** 1 Department of Ophthalmology and Visual Sciences, University of Illinois at Chicago, Chicago, IL 60612, USA; yteja@uic.edu; 2 Department of Microbiology and Immunology, University of Illinois at Chicago, Chicago, IL 60612, USA; ivolet2@uic.edu

**Keywords:** aptamers, SELEX, diagnosis, therapy, viruses

## Abstract

The use of short oligonucleotide or peptide molecules as target-specific aptamers has recently garnered substantial attention in the field of the detection and treatment of viral infections. Based on their high affinity and high specificity to desired targets, their use is on the rise to replace antibodies for the detection of viruses and viral antigens. Furthermore, aptamers inhibit intracellular viral transcription and translation, in addition to restricting viral entry into host cells. This has opened up a plethora of new targets for the research and development of novel vaccines against viruses. Here, we discuss the advances made in aptamer technology for viral diagnosis and therapy in the past decade.

## 1. Introduction

Aptamers are oligonucleotides or peptide molecules that bind specifically to a variety of targets, often inhibiting protein–protein interactions. While usually in the range of a hundred nucleotides, the most effective size is found to be around forty nucleotides. The term “aptamer” was first coined by the Szostak lab in the year 1990 and is derived from the Latin word “aptus”, which means “to fit”, and the Greek expression “meros,” which means “part” [1]. While natural aptamers exist in riboswitches, they are commonly created through the SELEX process. The acronym “SELEX” stands for the systematic evolution of ligands by exponential enrichment and was coined by the Gold lab [2]. In SELEX, large libraries of random sequence pools (10^13^–10^16^ copies) of ssRNA/ssDNA are progressively screened and selected, based on their ability to bind to the desired target, as well as their abilities to inhibit or activate various processes.

In this regard, various aptamers with applications ranging from catalysis to cancer therapy have come into existence. Their increased use and development have been noted for the detection and therapy of a variety of diseases, including cancer, diabetes, auto-immune diseases, bacterial and viral infections [3,4,5,6,7]. Of these, the diagnosis and therapy of viral infections via aptamers continue to generate great interest, owing to their immediate societal impact. Although the past decade has seen significant improvement in the field of immunology, pharmacology and microbiology, millions of people continue to be diagnosed with, treated for, suffer, and die every year from viral infections, especially due to the human immunodeficiency virus (HIV), hepatitis viruses, influenza viruses and herpes simplex virus (HSV), both type-1 and type-2.

Addressing this problem, aptamers have shown substantial efficacy in both diagnosis and treatment, with minimal or no side effects. In addition, aptamers do not encounter the issue of drug resistance that traditional antiviral medications inevitably run into. Owing to these exceptional qualities, numerous research papers on the diagnostic abilities, and both practical and potential therapeutic properties of aptamers have come into existence over the past two decades.

In this review article, we elucidate in detail the advancements that have been made in the past decade in the field of aptamers, specifically as they pertain to viral diagnosis and therapy. We also discuss the nature of these viral infections, by detailing their cellular and molecular machinery involved in causing infection. Furthermore, a comprehensive review of aptamers developed for each of these cellular mechanisms involved in viral infection is described. By reviewing the most recent forms of aptamer modifications implemented by researchers for improving their efficiency, we also delve into the recent, more advanced developments that have been reported in the field of aptamer detection and treatment for HIV, hepatitis, influenza and HSV. 

## 2. Human Immunodeficiency Virus (HIV) 

(HIV) is a member of the *Retroviridae* family, which causes acquired immunodeficiency syndrome (AIDS) in humans, a lethal disease that kills millions of people worldwide. The structure of HIV has been studied closely, and multiple treatment models have been presented in the past. However, with the advent of aptamer technology, scientists are adopting a multifaceted approach to curing this life-threatening disease that is safer than other alternatives. The molecular details of entry, including the added information on the structure of the virus, play a key role in designing the perfect aptamers for prevention, inhibition, and recovery from a viral infection. Here we briefly discuss the various receptors, proteins and organelles involved in viral infection, while highlighting those that have been used for the design of aptamers. 

### 2.1. HIV Structure and Entry

HIV structure, cell binding and host cell entry were described in detail in a 2012 report [8]. HIV, like most other viruses, typically consists of an outer envelope and inner core proteins. The envelope consists of an outer lipid layer containing an envelope protein (Env) that is a heavily glycosylated trimer of the surface glycoproteins, the gp120 and gp41 heterodimers [9]. These proteins are believed to be critical for host cell binding during viral infection. Inside the lipid layer, a coat of matrix proteins termed “p17” is present between the outer envelope and inner core [10]. The inner core consists of the capsule protein “p24” that encapsulates single-stranded HIV RNA, HIV-reverse transcriptase (HIV-RT), protease, ribonuclease, and integrase [11]. 

HIV famously infects CD4^+^ lymphocytes; however, monocytes, macrophages, and dendritic cells (which are also CD4^+^) have all been shown to get infected [11]. The process of virus-host binding involves the viral proteins gp120 and gp41, and the host receptors CD4, CCR5 and CXCR4. HIV is attracted toward the host cell; whether this is due to the negatively charged heparan sulfate proteoglycans on the host cell surface has been a matter of debate [8]. When in close range with host cells, HIV’s gp120 interacts with the host’s CD4 receptor, which is followed by an intricate series of steps involving the restructuring of viral gp120. Subsequently, in the host cell, CCR5/CXCR4 co-receptor binding occurs, which is thought to elicit the membrane fusion potential of viral Env, resulting in the successful binding of the virus to the host cell. The last step in the fusion process involves the hydrophobic gp41 fusion peptide that tethers the viral and host membranes, forming a fusion pore [12]. 

**Figure 1:**
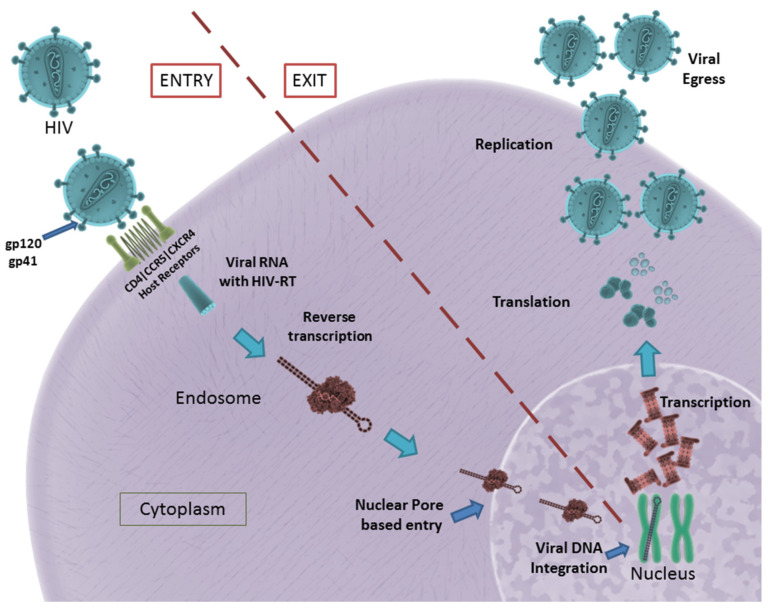
HIV entry and egress, as illustrated: Pictured are the important host receptors CD4, CCR5 and CXCR5. The viral glycoproteins gp120 and gp41 play an important role in attachment and entry; viral RNA is made and integrated into the host genome. After replication is facilitated, viral particles egress from the host cell. CC BY 4.0 license.

The formation of the fusion pore assists in the entry of viral RNA and HIV-RT into the infected cell. The HIV-RT is responsible for the conversion of viral RNA into DNA that is consequently integrated into the host genome [13]. The viral genome consists of nine genes, of which gag, pol and env encode for the structural proteins required for the formation of new virus particles. These genes are transcribed and translated inside the host cell, resulting in the formation and eventual assembly of new virus particles that egress from the host cell to infect other cells [14]. A schematic illustration of HIV entry and egress can be seen in Figure 1. 

In summary, gp120, gp41, viral Env, HIV-RT, CD4 receptor, CCR5 and CXCR4 co-receptors play a significant role in HIV infection. They remain potential targets for the development of aptamers in the successful inhibition of HIV infection. 

### 2.2. Aptamers in Anti-HIV Therapy

Owing to their conformational flexibility, RNA oligonucleotides have been used conventionally in the selection of aptamers for HIV therapy. However, multiple studies on DNA aptamers for the therapy/inhibition of productive HIV infection have also been described. In recent years, the focus was placed on developing aptamers for CD4, CCR5, gp120/gp41 and HIV-RT. Other notable aptamer targets include HIV-gag, thrombin, human cyclin T1, P24 antigen, aspartyl protease and nucleolin. 

Small interfering RNAs (siRNA) have gained a reputation for disrupting undesirable pathways, in turn inhibiting viral infections. However, siRNAs innately lack the ability to target the desired site and need to be transported via a molecular carrier. In 2011, Zhou et al. reported RNA aptamers targeting HIV’s viral gp120 [15]. By conjugating this 81-nucleotide-long aptamer to siRNA, their team was able to demonstrate inhibitory action on HIV infections. The reported RNA aptamer had a dissociation rate constant (K_d_) of 47.91 nM. Continuing this body of work, in 2013, the same group reported the in vivo delivery of the same aptamer-siRNA chimera using a chemically synthesized sticky bridge [16]. The sticky bridge facilitated the attachment of multiple siRNA chimeras to the same aptamer, as opposed to their previous model. They further reported the construction of three Dicer substrate siRNAs (DsiRNAs—presently available through IDT, Inc. 1710 Commercial Park, Coralville, Iowa 52241, USA) onto the sticky bridge that facilitates the effective delivery of three different siRNAs to an infection site, thereby inhibiting multiple mRNA nodes in the HIV transcription process. In 2015, they developed a new aptamer, through live-cell SELEX and high-throughput next-generation sequencing, for the CCR5 co-receptor [17]. This aptamer, when combined with siRNA chimeras, was able to effectively block HIV entry and neutralize R5 virus infection through internalization. These reports have been consolidated into a book chapter, published by the group, that provides new insight into the development of next-generation therapeutics for HIV without the obstacle of drug resistance. Interestingly, in 2011, Wheeler et al. reported a siRNA-based gene knockdown mechanism to inhibit HIV infection in vitro and in tissue explants [18]. However, they used a CD4-binding aptamer, as opposed to a gp120-binding aptamer. Later in 2013, a clinical trial on humanized mice revealed the effectiveness of inhibiting transmission during sexual intercourse using the developed aptamer-chimera vaginal gel [19]. 

In 2012, Khati et al. showed significant modifications to an earlier-reported gp120 RNA aptamer named UCLA1, which was a shortened synthetic version of its predecessor [20]. This aptamer was reported to tightly bind to the HIV gp120 (K_d_ = 0.15 nM) and elicited an IC_50_ (50% inhibitory concentration) in the nanomolar range. The group reported the synergistic effect of the aptamer alongside gp41- and CD40-inhibiting antibodies. UCLA1-based protection against HIV-mediated cardiomyopathy (HIVCM) was reported by [21]. They suggested that UCLA1 protects cardiomyocytes from caspase-mediated apoptosis directly by binding to HIV-1 and indirectly by preventing the infection of monocyte-derived macrophages. In 2015, the group reported a whole viral SELEX against HIV-1 subtype C to generate aptamers against all surface proteins of HIV [22]. The group not only isolated aptamers that bind to gp120 and gp41 but also some aptamers that bound to neither but that were able to elicit an inhibitory action on the HIV infection. Additional studies will need to be conducted to further understand the neutralizing properties of the isolated aptamers. 

**Figure 2:**
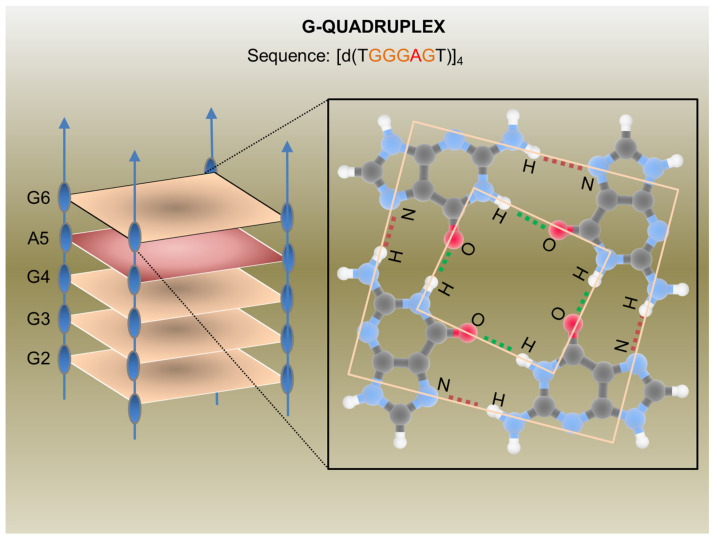
G-Quadruplex aptamer detailed in a report by Virgilio et al.: Schematic representation of the anti-HIV G-Quadruplex aptamers detailed in a report by Virgilio et al. [35] (on the left) and an example of a G-tetrad showing H-bonds (on the right). CC BY 4.0 license.

In the last decades, many synthetic G-rich oligonucleotides have been identified as promising anti-HIV candidate drugs [23,24]. Briefly, guanine-rich sequences often form hierarchical structures called G-quadruplexes which have unique target binding abilities (Figure 2).

The first G-quadruplex-forming oligonucleotide identified as a potent anti-HIV agent was the 8-mer phosphorothioate TTGGGGTT (ISIS 5320) [25], which exhibited the inhibition of HIV-1 at sub-micromolar concentrations. ISIS 5320 forms a tetramolecular parallel-stranded G-quadruplex, which is able to bind the V3 loop of the envelope glycoprotein gp120 and inhibit virus adsorption and cell fusion (Figure 1). Following these studies, Hotoda and coworkers have investigated a large number of G-rich oligonucleotides, some of which offer promising anti-HIV activity targeting HIV-1 entry through gp120 binding [26]. They selected the 6-mer d(5′TGGGAG3′), successively identified as “Hotoda’s sequence” (Figure 2), as the lead sequence, which turned out to be active against HIV-1 at submicromolar concentrations only when conjugated at the 5′-position with bulky aromatic moieties [27]. Subsequent detailed studies on the 6-mer d(5′TGGGAG3′), chosen as a useful model system, better elucidated the structure–activity relationships of G-quadruplex-forming oligonucleotides endowed with antiviral activity [28,29,30].

In 2014, Romanucci et al. prepared a number of novel analogs bearing different hydrophobic tails at the 5′-ends of Hotoda’s sequence for the inhibition of HIV [31]. According to the study, the developed aptamers had low cytotoxicity, high anti-viral activity, good structural stability, and elevated resistance in human serum. Later, this group reported the development of a biomolecular G-quadruplex, with an HEG loop as an inversion of the polarity sites 3′-3′ or 5′-5′ and aromatic residues conjugated to 5′-end, which possessed greater thermal stability than its predecessor [32]. However, no increase or decrease in anti-HIV activity was reported. By chemically connecting the 3′- and/or 5′-ends of four d(5′TGGGAG3′) strands, thus obtaining bunchy oligonucleotides, unimolecular G-quadruplex structures with interesting anti-HIV properties were realized [33,34]. In 2012, Virgilio et al. gave a detailed report on the structure of the anti-HIV G-quadruplex-forming oligonucleotide TGGGAG and its analogs using various biochemical and structural studies [35]. They proposed the presence of an A tetrad in the G-quadruplex. 

**Figure 3:**
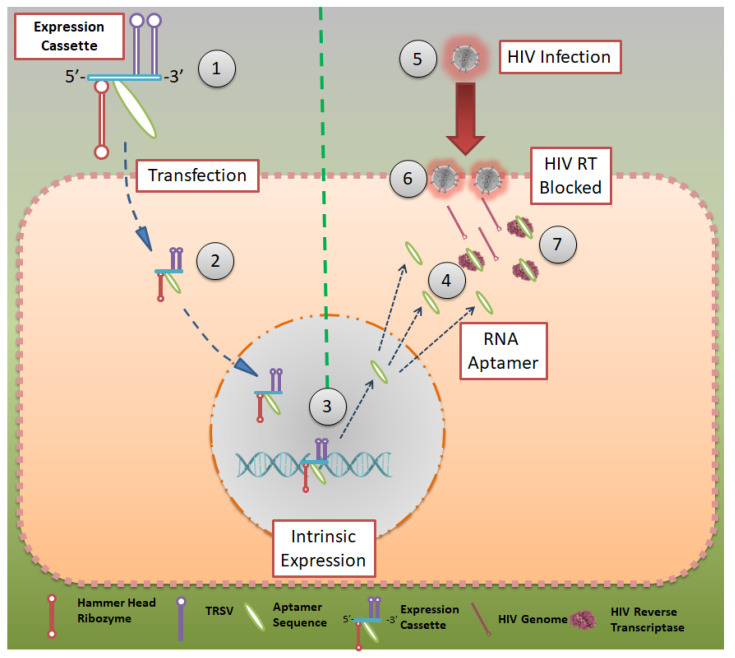
Aptamer expression cassette: Expression cassette modeled after HIV-RT that can be produced in intracellularly modified cells. Hammerhead ribozymes that are tertiary stabilized with self-cleavage activity may be expressed in infected cells. CC BY 4.0 license.

Although all the techniques and aptamers mentioned above are effective in reducing HIV infections, they do not elicit immunity in an individual who is being treated with them. In 2012, Burke et al. reported a robust model to inhibit HIV infection, where HIV-RT-inhibiting RNA aptamers were produced intracellularly by modified cells [36]. They showed that aptamers that bind to the HIV-RT region could potentially inhibit productive replication and thus HIV infection (Figure 3). Using tertiary-stabilized hammerhead ribozymes with enhanced self-cleavage activity, aptamers were flanked into an expression cassette to be expressed in an infected cell. This significantly increased aptamer accumulation in viral and cellular compartments, neutralizing any HIV-RT present in the cell.

In 2013, the group showed a high-throughput sequencing (HTS)-based SELEX process in which different populations of RNA were evaluated for their binding capacity to the HIV-1 virus [37]. HTS analysis was used to reveal the structural and functional details of various converged motifs that may otherwise be obscured by simpler consensus descriptions. HTS was used to distinguish 181 clusters determined to be capable of forming a conserved UCAA element in a non-pseudoknot motif [38]. This was established by screening 100 full-length and 60 truncated aptamer transcripts for their F1Pk and F2Pk pseudoknot signature motifs. In 2014, this group reported a novel testing/screening model for RNA aptamers without the necessity of serial passages of HIV in aptamer-expressing cell lines [39]. The expression cassette mentioned earlier was used as a screening model, where a variety of aptamer sequences could be added to the cassette and later inserted into the cell line to help them express the same. The aptamer-expressing cell lines could then be tested for HIV-RT inhibition to screen for the most suitable aptamers. 

### 2.3. Aptamers in HIV Detection

Although non-conventional HIV-detection kits have not hit mainstream markets, they harbor an enormous potential to reduce the burden on diagnostic labs and change the landscape of the patient population visiting diagnostic labs if they become available. Aptamer technology represents one such way to improve the diagnostic capability regarding HIV infections. 

Multiple models currently exist for the detection of HIV infections, of which the optical and fluorescent models remain popular. Rahim et al. [40] developed an aptamer for detection of the HIV trans-activator of the transcription (HIV-tat) peptide, which was then immobilized onto a diamond surface. The diamond surface formed part of a field emission transistor (FET) that was used for the potentiometric detection of HIV. The group showed that the detection range was in the picomolar range (1 pM to 1 μg/mL). The group followed this up with a report on the diamond aptamer FET in real sample scenarios, with detection at 1nM and a higher standard of reliability than current conventions [41]. 

**Figure 4:**
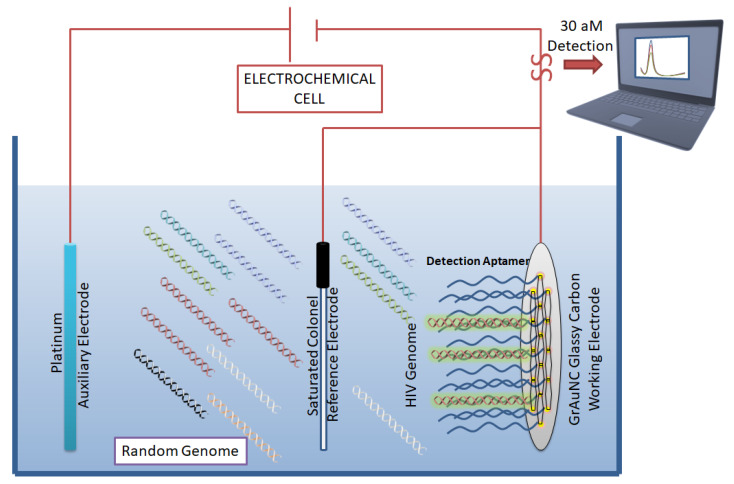
Aptamer-based biosensor for HIV detection: Graphene/gold nanocluster glass carbon electrode developed as a biosensor for the detection of HIV-DNA by Wang et al. They report the biosensor’s potential for diagnostic analysis of human serum. CC BY 4.0 license.

In 2011, Liang et al. developed a direct visualization technique by conjugating fluorescent beacons to HIV-RT targeting aptamers [42]. Another fluorescent-based detection technique was developed by Kim et al. [43], in which double-stranded and dual-anchored BHQ1-attached aptamers were conjugated to reduced graphene oxide nanosheets (Figure 4). Interferon-γ was detected in the low picomolar range using this technique. This system was also tested for the detection of non-HIV targets, such as interleukin-2 and tumor necrosis factor-α. This module was able to rapidly quantify HIV infection in serum samples derived from HIV-infected individuals in less than 10 min. 

In an ergonomic model, Niedzwiecki et al. demonstrated that by using nanopores that are smaller than the largest aptamer-target conjugate, one can screen for successful aptamers and determine their K_d_ values simultaneously [44]. In this study, the authors report the label-free determination of K_d_ between nucleocapsid protein 7 of HIV and the stem-loop 3 of an RNA aptamer. The nanopores were 6 nm in diameter and were made of silicon nitride. In a separate study, an atomic-force microscope (AFM) was used to carry out direct and label-free detection of gp120 HIV type-1 envelope glycoprotein as a target protein [45]. The method used anti-gp120 aptamers immobilized on the AFM chip to count gp120/aptamer complexes that formed on the chip surface. The detection limit was seen in the range of 0.8 nM to 8 pM. However, the lowest detection limit of 30 aM was reported by Wang et al. [46], who used a graphene/gold nanocluster modified glassy carbon electrode for the detection of HIV-DNA. They proposed that the developed biosensor was promising for the diagnostic analysis of human serum (Figure 4). 

Various other aptamers that bind to the CCR5 and gp41 regions have been reported and are noted in Table A1 [39,47,48,49,50,51,52,53,54,55,56,57,58,59,60,61,62,63,64,65,66,67,68,69,70,71].

## 3. Hepatitis Virus (HBV, HCV) 

Viral hepatitis is caused by 5 well-known viruses, namely hepatitis A virus (HAV), hepatitis B virus (HBV), hepatitis C virus (HCV), hepatitis D virus (HDV) and hepatitis E virus (HEV). Of these, HBV and HCV are the most studied with respect to their structure and mode of entry into a host cell. Despite the similarity in their names, HBV and HCV belong to two different virus families, with profoundly distinct features. 

### 3.1. HBV Structure and Lifecycle

HBV belongs to the *Hepadnavirus* family, which consists of a spherical, partially double-stranded DNA inside an icosahedral nucleocapsid core protein. Similar to HIV, HBV contains DNA polymerase, which has reverse transcriptase activity. The outer lipid membrane consists of three kinds of HBV surface antigens (HBsAg)—“S”, “M” and “L” HBsAg, which are important for virus-cell fusion by targeting the surface receptors of the host cell [72,73]. 

HBV attaches to host hepatocytes through the sodium/bile acid co-transporting peptide SLC10A1, with the help of HBsAg. This is followed by a clathrin-mediated endocytosis step that allows for the internalization of the virus. HBV DNA and core proteins are then released into the cytoplasm and are then transported by the cellular machinery into the nucleus, through a nuclear pore [74]. 

HBV DNA undergoes a transformation from partially double-stranded to a covalently closed circular double-stranded DNA (cccdsDNA) inside the nucleus. At this stage, cccdsDNA is converted into a long mRNA using host RNA polymerase, which is then transported into the cytoplasm for translation. The viral mRNA not only codes for core proteins and other structural components but also for the genome of the newly born virus. The virion P protein, which is known to be synonymous with reverse transcriptase, is responsible for the synthesis of viral DNA [75]. 

### 3.2. HCV Structure and Lifecycle

**Figure 5:**
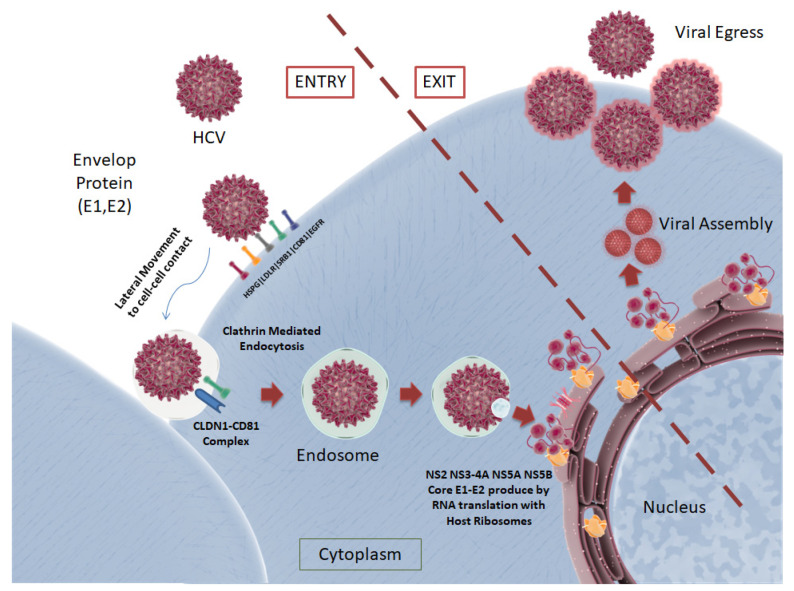
The HCV lifecycle depicted: Two surface proteins, E1 and E2, present on the viral envelope play a role in the attachment to host cell receptors LDLR, HSPG, SRB1, CD81 and EGFR. RNA replication with host ribosomes takes place, followed by viral particle assembly and egress. CC BY 4.0 license.

Unlike HBV, HCV is a positive-strand RNA virus that belongs to the *Flaviviridae* family. It is known to showcase two surface glycoproteins, “E1” and “E2”, on its envelope that envelop a single strand of the viral RNA, encompassed by core proteins [76]. The complete HCV lifecycle is depicted in Figure 5. 

HCV circulates in the blood, where it encounters the basolateral surface of hepatocytes. The surface E1 and E2 glycoproteins attach to the surface receptors, which assist in the entry of the virus into the cell. Evidence is also available that HCV hybridizes with lipids and cholesterol in the blood to form “lipo-viral particles” (LVP) that give HCV immunity against antibody neutralization before entering the host cell [77]. Five surface receptors, namely, CD81, scavenger receptor class B member 1 (SRB1), claudin 1 (CLDN1), occludin (OCLN) and the cholesterol absorption receptor Niemann–Pick C1-like 1 (NPC1L1) are required for suitable attachment of the virus to the cell surface. HCV is known to interact with low-density lipoprotein receptors (LDLR) and the glycosaminoglycans present on the heparan sulfate proteoglycans (HSPG), which remove the lipid components from the LVP [77]. This induces structural changes to E2, enabling CD81 binding. Post-CD81 binding, the HCV particle then moves laterally to the cell–cell contact, where CD81 interacts with CLDN1. Similar to HBV, HCV is then internalized by clathrin-mediated endocytosis, where the endosome contains a low-pH compartment [78]. This prompts the fusion of the viral capsid via NPC1L1 and OCLN, releasing viral RNA into the cytoplasm [79]. 

Via translation of the viral RNA, a polyprotein is produced that is cleaved by both viral and cellular proteases. Each polypeptide chain of the polyprotein encodes for structural proteins, core proteins, glycoproteins and non-structural (NS) proteins. Non-structural proteins, namely, p7, NS2, NS3, NS4A, NS5A and NS5B, are not incorporated into the virus but help coordinate HCV replication, viral RNA synthesis and the assembly of virus particles. HCV particles are formed by budding into the endoplasmic reticulum (ER), followed by the sequential assembly of glycoproteins, core proteins and the viral RNA. It has been shown that post-assembly, HCV particles follow a series of subcellular compartments (secretory pathway) to the exit cells [80]. 

### 3.3. Aptamers for Hepatitis Virus Therapy

From the collected data, it was evident that DNA aptamers were predominantly used for the treatment of HBV and HCV. It is also interesting to note that most of the research conducted in recent years is an extension of previously reported aptamers, as opposed to novel oligonucleotide sequences. This could be due to well-defined, commercially available aptamers through reputed sources. It is also worth noting that studies have predominantly concentrated on HCV diagnosis and therapy rather than HBV or other hepatitis viruses. 

In 2011, Feng et al. demonstrated a proof-of-concept by constructing an RNA aptamer that was able to bind and suppress the HBV P protein [81]. As mentioned previously, the HBV P protein is crucial for viral RNA replication and the subsequent progeny. In this study, they reported a first-in-class inhibitor of viral RNA replication through a 29-nucleotide RNA aptamer that was screened with the SELEX process using a recombinant truncated HBV P protein. A review article, detailing the prospects of inhibiting the post-transcriptional regulation of HBV genes, by Chen et al. explains the same finding in further detail [82]. In 2014, Zhang et al. reported a DNA aptamer that was commercially selected and synthesized using the SELEX process against the HBV core protein [83]. The aptamer showed the complete inhibition of HBV viruses in HepG2.2.15 cell lines, by disrupting the assembly of the viruses. In 2015, Orabi et al. reported the development of DNA aptamers that specifically bind to HBV capsid but not to the isoleucine 126A matrix domain [84]. This was achieved by targeting aptamers to the capsid proteins while counter-selecting them against isoleucine 126A. Although the developed aptamers were able to bind to the viral capsid, their inhibitory action was low. The low half-life time (2 h) in cells was determined to be the limiting factor influencing the incomplete inhibition. 

Berzal-Herranz et al. demonstrated the development of RNA aptamers for the inhibition of the cis-acting replication element (CRE) located on the 3′ end of the NS5A coding region [85]. They showed an 80% reduction in viral infection in Huh-7 cell cultures. They also developed an RNA aptamer for the inhibition of the highly conserved internal ribosome entry site (IRES) on the 5′ untranslatable region of the HCV genome [86]. They proposed that targeting molecules to these regions would completely disable the production of viral proteins, as viral translation greatly differs from cellular translation processes. In 2013, the same group developed RNA aptamers that were able to target conserved regions of the viral RNA which coded for the NS5A RNA polymerase [87]. They identified multiple aptamers that could inhibit viral replication by approximately 50% at concentrations close to 0.5 μM. 

A study by Lee et al. reported the selection of RNA aptamers and their consequent 2′-hydroxyl, 2′-fluoropyrimidine modification against the NS5B replicase [88,89]. By blocking this enzyme, the study effectively showed the inhibition of HBV viral replication without any damaging off-target effects or escaping mutant generation. In 2015, this aptamer was further modified by cholesterol conjugation, resulting in better bioavailability in a mouse model [90]. A review article [91] by the group reports various other aptamers that are available and the prospects of nucleic acid-based therapeutics for HCV. 

**Figure 6:**
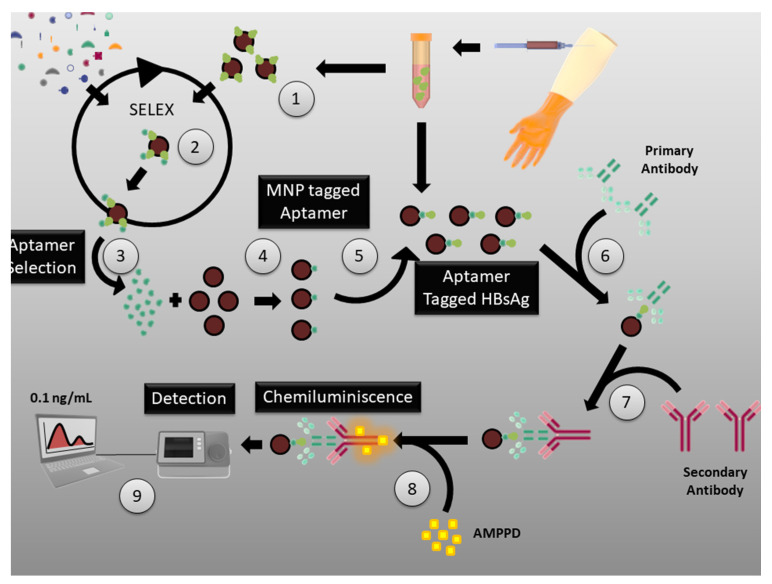
SELEX process for the generation of aptamers against HCV: E1 and E2 proteins of HCV used as targets in SELEX to generate aptamers against virus particles that bind to host cells. CC BY 4.0 license.

In 2013, Yang et al. reported the production of aptamers that target surface E1 and E2 proteins of HCV [92]. Purified forms of the E1 and E2 proteins were used as SELEX targets to generate the desired aptamers. The group suggested that these aptamers were able to decrease infection through the inhibition of the virus particles that bind to host cells. In 2015, Delaviz et al. also reported the development of DNA aptamers against E1 and E2 surface proteins [93]. These aptamers were conjugated to magnetic nanoparticles and, through magnetic separation, productive infection in the blood serum was selectively isolated and removed (Figure 6). 

### 3.4. Aptamers for Hepatitis Virus Diagnosis

A competitive binding assay using RNA aptamers was developed by Suh et al. [94] that helped them to detect the HBV surface antigen in samples without any pre-treatment. They claim that their protocol is 40 times more sensitive than the commercially available Abbott Architect assay for HBV surface antigen detection. Similarly, in 2015, Xi et al. reported a chemiluminescence assay with a detection limit of 0.1 ng/L for the detection of the HBV surface antigen [95], using DNA aptamers. 

Using an Octet optical biosensor, Roh et al. were able to detect the viral NS5B protein with the help of RNA aptamers [96]. The biotinylated RNA oligonucleotide optical biosensor was able to detect target proteins as low as 700 pg/mL. Another rapid yet specific lateral-flow strip for the detection of HCV was developed by Wang et al. using a DNA aptamer [97]. The aptamer was able to target HCV core proteins with a detection limit of close to 10 pg/mL. Similarly, an enzyme-linked aptamer-sorbent assay (ELASA) for the detection of the HCV E2 protein was developed by Park et al., which had a detection limit of 0.8 nM [98]. A full list of aptamer for hepatitis virus diagnosis and therapy are listed in Table A2 [99,100,101,102,103,104,105,106,107,108,109,110].

## 4. Influenza Virus

Influenza viruses belong to the *Orthomyxoviridae* family and are sub-divided into three major types: influenza types A, B and C viruses. Of these, A and B primarily cause infections in humans and are known to cause flu pandemics around the world. All three influenza virus types have been thoroughly studied and their life cycles are well documented. To address the significant public-health cost and threat caused by these viruses, aptamer technology has seen rapid progress to provide cost-effective solutions to influenza infections. Here, we will review the structure and life cycle of the influenza-A virus and its sub-types, while highlighting some key anti-influenza aptamers. The information regarding the structure and lifecycle of the influenza-A virus can be found in much greater detail in the Elsevier article report on rapid-reference influenza [111]. 

### 4.1. Structure and Lifecycle of Influenza-A Virus

**Figure 7:**
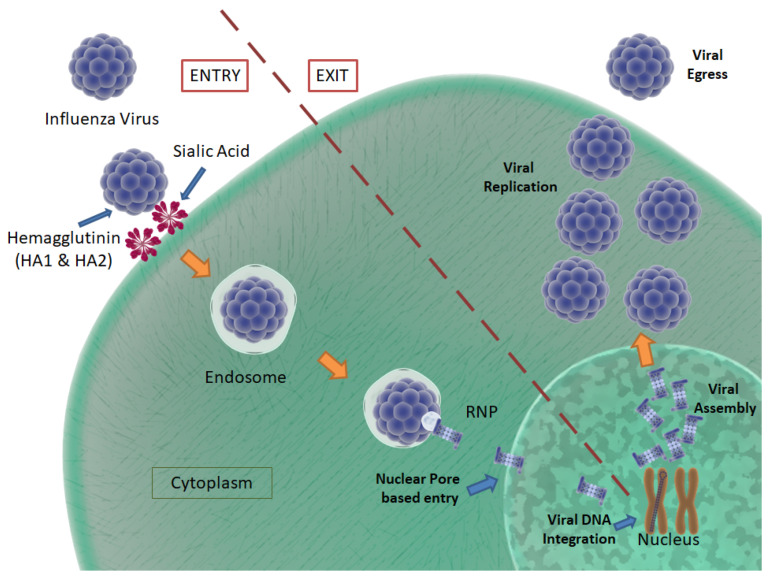
Influenza virus life cycle: Influenza virus entry, integration, assembly and egress, as pictured. The viral envelope is composed of HA and NA proteins. The HA proteins, HA1 and HA2, play a role in the fusion process of the virus T host cell. NA cleaves bonds between the host surface receptors and assists in viral egress. CC BY 4.0 license.

The influenza-A virus is characterized by the presence of two major surface glycoproteins, namely, “hemagglutinin” (HA) and “neuraminidase” (NA). There are 16 HA subtypes and 9 NA subtypes. Based on the nature of these proteins, various influenza-A virus subtypes have been discovered and, of those, only H1N1, H2N2 and H3N2 viruses are considered mortally harmful to humans. The complete lifecycle of a typical influenza virus is depicted in Figure 7. 

The influenza virus consists of 8 negative-sense RNA segments, nucleoprotein, and polymerase inside a lipid membrane envelope, which is coated by M1 proteins on the inner surface and M2 proteins on the outer surface. As mentioned earlier, the envelope is also encompassed by rod-shaped HA and mushroom-shaped NA, present on the outer surface in a 4:1 composition ratio. HA consists of two major subunits, namely, HA1 and HA2, which are bound by disulfide bonds. The HA2 subunit consists of a conserved hydrophobic amino acid chain, known as a fusion-peptide, which conducts the membrane fusion process between the virus and host cells. Conversely, NA has enzymatic activity that cleaves the bonds between surface receptors and the virus, assisting in viral egress [112]. 

The influenza virus enters a host body through both the enteral and parenteral routes. When it encounters the epithelial cells of the mucous membrane, the virus attaches to sialic acid residues, via HA binding [113]. Post-binding, receptor-mediated endocytosis is activated that allows the uptake of the virus into the host cell. The viruses are encapsulated in the host plasma membrane in the form of endosomes, where the virus fuses with the endosome to attain access to the host cell’s cytoplasm. As is synonymous with HCV, the low pH of the endosomal compartment is known to cause a fusion process between the viral envelope and the endosomal membrane. Here, HA2 plays a vital role in binding with the endosomal membrane, eventually fusing both together to create an endosomal pore through which the viral RNA, nucleoprotein and polymerase are released into the cytosol [114]. 

The viral genome traverses through the cytosol to enter the host nucleus, where the viral nucleoprotein transcriptase (consisting of PB1, PB2 and PA) converts the negative-sense viral RNA to messenger RNA (mRNA) [112]. The mRNA is then transported into the cytosol, where it is transcribed to form structural proteins, such as HA, NA and M2. The mRNA also serves as the template for the creation of negative-sense viral RNA for the new virus particles. Viral proteins are translocated to the endoplasmic reticulum, where they are glycosylated before they are carried to the Golgi apparatus. Here, the M2 protein has been shown to block premature HA unfolding due to the acidic nature of the trans-Golgi network [111]. Viral proteins and the genome are then transported to the host plasma membrane, starting the budding process. After the completion of the budding process, the viruses are still attached to the plasma membrane through the binding of sialic acid residues. They are enzymatically cleaved by NA to dissociate them from the plasma membrane, releasing new virus particles into the apical side of the epithelial cells [115]. 

### 4.2. Aptamers for the Treatment of the Influenza Virus

Most studies done on developing aptamers against influenza viruses have concentrated on H1N1, H3N2, H5N1, H5N2, H9N2, and PERTH sub-strains. It is worth noting that DNA aptamers are more commonly used in recent years, as opposed to RNA aptamers. The common target in SELEX (for anti-influenza aptamer isolation) was hemagglutinin, specific for the influenza virus. 

RNA aptamers against the hemagglutinin of the H1N1 virus were developed by Gopinath S.C.B. and Kumar P.K.R. in 2013 [116]. The aptamers showed significantly low K_d_ values, which was indicative of their use as effective inhibitors of viral entry. Musafia et al. reported a DNA aptamer—BV02—that had high specificity toward hemagglutinin [117]. They developed a quantitative structure–activity relationship (QSAR) tool for the effective determination of 2D structural motifs of the target and aptamers. This helped them determine that the activity/binding affinity of the aptamer was less specific to the sequence and showed more specificity to the structure. Using the QSAR tool, they were able to predict with 89% accuracy the binding affinity between a known aptamer and the desired target. Wongphatcharachai et al. developed neutralizing DNA aptamers against the H3N2 virus by targeting the H3 hemagglutinin [118]. The strongest contender for binding had the lowest K_d_ of 3.7 nM and was able to bind to the virus in serum samples extracted from clinical patients. 

In 2013, a G-Quadruplex-based DNA aptamer against the NS-1 protein of the influenza H5N1 virus was developed [119]. While NS-1 inhibits the host’s innate immune response, the DNA aptamer was able to induce the production of IFN-β, leading to the effective suppression of the NS-1 protein and viral replication, without affecting host cell viability. An RNA aptamer against the highly pathogenic avian influenza H5N1 virus was developed by Suenaga and Kumar [120]. This aptamer showed selective binding to viral H5 rather than H7 hemagglutinins. In a 2015 study, DNA aptamers were developed against the H5N1 PA subunit, which actively takes part in RNA polymerase and endonuclease activity [121]. These aptamers have low K_d_ values as well as an IC50 concentration as low as 10 nM, making them very exciting additions to the field. 

Two RNA aptamer studies conducted in 2014, targeting the hemagglutinin of the H5N2 virus, were reported by Kim et al. [122,123]. They reported aptamers that bind to the glycosylated HA1 domain of hemagglutinin and showed complete anti-viral activity with no detected reduction in cell viability. These two DNA aptamers, developed by Choi et al. [124] and Zhang et al. [125], showed potent inhibition of the virus in vitro against the H9N2 virus. While both the groups targeted the HA component of the virus, Zhang et al. used capillary electrophoresis-based SELEX for the selection of successful aptamers. 

### 4.3. Aptamers for the Detection of the Influenza Virus

Detection of the influenza virus during the past five years has predominantly concentrated on the H5N1 virus. This can be attributed to the rising numbers of reported deaths caused by the highly pathogenic H5N1 virus in many parts of Asia and the Middle East. Avian influenza is still a persistent public health threat in many Asian countries. 

Of the various sensors used for the detection of the H5N1 virus, electrochemical impedance-based sensors were the most used. This could be due to the ease of building one in a small amount of time. Lui et al. have reported multiple studies on the detection of the H5N1 virus [126,127,128,129,130,131]. While earlier reports by the group studied the process of SELEX for developing aptamers against hemagglutinin [126,127,128,129], recent updates have reported the use of the entire virus as a target [130,131]. 

All the studies used DNA as the nucleic acid of choice. Using a surface plasmon resonance-based setup (SPR), biotinylated aptamers were used for the rapid detection of a virus. The detection limit in this report was noted as 0.128 hemagglutinin units (HAU). A similar study that used quartz crystal monitors instead of an SPR setup showed a lower detection limit of 0.0128 HAU. In a different study, an SPR-based SELEX process was used to elucidate the binding affinity of the aptamer in targeting the hemagglutinin of H5N1. The group proposed this technique as a potential novel system for the screening of aptamers. The same group showed a detection limit of 0.0004 HAU, using an enzymatic sandwich assay with concanavalin A, glucose oxidase, and Au nanoparticles. 

Using the models described above, another study described a detection methodology that used an aptamer bio-nanogate that could selectively respond to target molecules and control enzymatic reactions for electrochemical measurements. The setup had a detection limit of 2–9 HAU and the capacity to sense within 1 h of activation. In a follow-up report, the same group revealed a microfluidic setup, consisting of biotinylated aptamers that could sense H5N1 viruses, with a detection limit of 0.0128 HAU within 30 min of activation. In 2011, Liu et al. revealed an electrochemical sensing assay with a detection limit that was the lowest of all the detection setups mentioned earlier [132]. The multi-wall carbon nanotubes, polypyrrole nanowires, gold nanoparticles and DNA aptamer-based setup had a detection limit of 43 pM for H5N1-specific gene sequences. In an article by Diba et al., this detection limit was exceeded by demonstrating detection up to 100 fM using an amperometric sensor [133]. The sensor relies on the formation of a complex consisting of gold nanoparticles and the DNA aptamer/H5N1/antiH5N1-alkaline phosphatase. In contrast to these developments in aptamer technology, a glycan-based impedimetric sensor utilized in 2016 had a detection limit of 5aM—claiming to be the most sensitive detection model, compared to current antibody or aptamer-based detection systems [134]. 

A G-Quadruplex DNA aptamer-based SPR sensor was also reported in 2015, which was able to detect H1N1 hemagglutinin with a detection limit in the nM range [110]. It was the only study that reported a G-Quadruplex DNA aptamer for H1N1 influenza sensing/detection. 

In 2011, Negri et al. used a surface-enhanced Raman (SERS)-based aptamer assay for the detection of viral nucleoproteins [135]. The study showed a detection limit of 0.1 μg/mL, using a 22-nucleotide-long DNA aptamer. The same group demonstrated a direct optical detection method of viral nucleoproteins, using a multi-variant anti-influenza aptamer [136]. This SERS-based method had a detection limit of 1 μg/mL and was validated by atomic force microscopy studies. A full list of aptamers that help in the diagnosis and therapy of influenza virus infection are listed in Table A3 [134,137,138,139,140,141,142,143,144,145,146].

## 5. Herpes Simplex Virus (HSV)

Herpes simplex virus (HSV) is a double-stranded DNA virus that belongs to the *Herpesviridae* family. HSV has two serotypes, namely, HSV-1 and HSV-2, which predominantly cause orofacial and genital lesions, respectively. It is estimated that upwards of 70% of American adults suffer from HSV infection, and it is the main cause of infectious blindness in the USA. HSV is infamous for recurring infections leading to herpetic lesion outbreaks, owing to a significant decrease in host immunity or high stress levels, which makes the virus a serious global public health threat [147,148,149].

### 5.1. HSV Structure and Lifecycle

**Figure 8:**
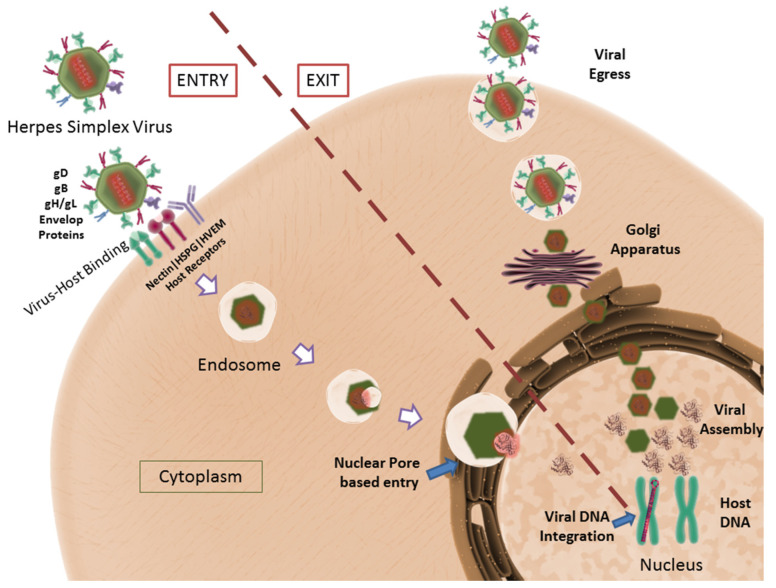
HSV life cycle depicted: The viral proteins gD, gB, gH/gL facilitate virus–host binding. The host receptors, Nectin, HVEM and HSPG have been shown to assist viral entry. The transcription and translation of viral genes are followed by viral egress. CC BY 4.0 license.

HSV consists of single linear double-stranded DNA, enclosed within an icosahedral capsid that consists of the four capsid proteins—VP5, VP26, VP23, and VP19C [150]. The capsid is surrounded by a protein-rich layer containing tegument proteins, which are known to regulate the transcription of immediate-entry genes. The most documented tegument proteins include VP16 and VP22, which regulate and stabilize viral transcription and proteins, respectively [151,152]. The tegument proteins are encapsulated inside a triple-layered lipid membrane that forms the envelope for the virus. The viral envelope is covered with four distinct surface glycoproteins, namely, gB, gC, gD and a gH/gL heterodimer [153,154]. These surface glycoproteins facilitate the attachment of the virus to host cells and the subsequent fusion process. A consolidated depiction of the HSV lifecycle is represented in Figure 8. 

Viral gB and gC surface glycoproteins interact with Heparan sulfate proteoglycans (HSPGs) to facilitate the viral attachment to the host cell membrane [155]. Using these proteins, the virus has been shown to surf along the large surface area of filopodia to reach the cellular surface, where the receptors required for virus fusion are present. When in close proximity to the cell surface, viral gD interacts with three distinct host surface receptors for fusion and entry, namely, herpes virus entry protein A (HVEA), 3-O-sulphated heparan sulfate, and nectin [155]. After attachment, viral gD binds to nectin and undergoes a conformational change, to interact with viral gB and gH/gL, ultimately leading to the fusion of the viral envelope to the host cell membrane. Upon fusion, the viral capsid and the tegument proteins are released into the host’s cytoplasm [156]. 

The viral capsid follows a trans-microtubule network to reach the nuclear pore, where the viral DNA is injected into the nucleus [157]. Simultaneously, tegument proteins enter the cytoplasm of the host to prepare for viral transcription and protein stabilization. Once inside the nucleus, the viral DNA can either initiate a lytic or latent infection cycle. In lytic infections, viral DNA is replicated for further processing, while in latent infections, the DNA is integrated into the host cell genome [158]. Upon initiation of the lytic cycle, immediate early (IE) genes (ICP0, ICP4, ICP22, ICP27, and ICP47) are expressed with the help of VP16 [159]. The ICP4 and ICP27 gene products initiate the expression of early (E) genes that help to regulate the replication/production of viral DNA. To conclude the cycle, late (L) genes are expressed that primarily code for the structural proteins required for viral assembly [160]. However, in the case of latent infection, only latency-associated transcripts (LAT) are transcribed, the function of which is still under investigation [161,162]. 

Closely regulated expression and transcription of all genes result in the replication and assembly of the viral proteins and genome to form virus particles. A significant contribution in HSV studies was conducted by our group, showing that heparan sulfate, required for the attachment/fusion of the virus to the host cell, is also responsible and implicated in holding newly budded virus particles to the host cell membrane. Heparanase, a host enzyme, is required and upregulated in infected cells, which cleave surface heparan sulfate, aiding viral egress from the cells [163]. 

### 5.2. Aptamers for HSV

There are two interesting studies that have been published in the field of aptamers treating HSV infections. While one study considered aptamers for HSV-1, the other study explored aptamers targeting HSV-2. Both the reports used RNA aptamers in their studies. 

Potent aptamers with an IC50 range of 20–50 nM were reported to block infection pathways that were dependent on both major entry receptors, Nectin1 and HVEM of HSV-2, by Moore et al. in 2011 [164]. The study showed the use of a robotic SELEX process to screen for aptamers against the gD protein of HSV-2. The screened aptamers were able to bind to their target with the same binding affinity as a gD antibody. Chinese hamster ovary (CHO) and Vero cell lines were infected with HSV-2 in both the presence and absence of the aptamers and showed a significant reduction in viral replication in the case of aptamer treatment. 

In a similar study, Gopinath et al. developed RNA aptamers against the gD of HSV-1 in 2012 [165]. The initial 113-nucleotide RNA was sequentially reduced to 45 nucleotides while keeping its K_d_ value approximately constant. The final aptamer had a K_d_ value of 40 nM, with an IC50 value of 0.8 μM. This was done by closely monitoring the structural changes that occurred with the removal of non-essential domains from the aptamer. 

Our group has reported the use of a DNA aptamer for the treatment of an HSV-1 infection in the eye [166]. The aptamer used the same sequence of the RNA aptamer that Gopinath et al. used to detect the HSV-1 gD, but in its DNA form. The aptamer has a K_d_ of 50 nM with an IC50 of 2 µM when used in PBS solution. This is the first and only DNA aptamer that has been studied in detail with respect to its therapeutic efficacy, both ex vivo and in vivo. This body of work has the potential to change the current landscape of perspectives on topical antivirals in treating HSV-1 keratitis. Topical antivirals have lower systemic toxicity-based side effects, so using anti-inflammatory and biocompatible drugs such as DNA aptamers can improve a disease prognosis significantly when it comes to HSV-1 keratitis.

A list of aptamers that have been used for the treatment and diagnosis of other viruses, such as apple stem pitting virus, coronavirus, norovirus, etc., have been tabulated in Table A4 [167,168,169,170,171,172,173,174,175,176,177,178,179,180,181,182,183,184,185,186,187,188,189,190,191,192,193,194,195,196,197,198,199,200,201,202,203,204,205].

## 6. Discussion

### 6.1. Role of Modern Technologies in Developing Aptamers

As aptamers are characteristically short nucleic acid sequences, any development in the field of nucleic acid research directly influences them. In this regard, the advent of newer and more sophisticated DNA sequencing systems has greatly helped the production of aptamers itself. The SELEX process heavily relies on understanding the repeatability of an oligonucleotide sequence in successive runs. With every successive round of selection, the aptamers that are bound to the target molecule are produced in higher quantities. Prior to 2011, the selected aptamer sequences would then be cloned into bacteria, and the colonies with the highest repeatability would be taken for further consideration. With the advent of next-generation sequencing techniques, a lot of these processes require no cloning or post-selection processing. This has hugely affected aptamer development globally in the past decade. In the last five years, the price for evaluating the sequence of a human-sized genome has fallen further to half its price, resulting in more researchers opting for this technique over older ones. Additionally, the production/commercialization of instruments that have next-gen sequencing capabilities have given researchers access to new tools that they did not have a decade ago [206].

As SELEX comprises a repeated selection process spanning anywhere between 10 and 20 cycles, robotic protocols with minimal human intervention have facilitated an error-free, simple and extremely efficient evolution of aptamers [207]. Combined with next-gen sequencing systems, these robotic protocols have reduced the SELEX timeline (including post-selection modification) from 3 to 6 months to just 6–8 weeks [208]. The list of companies providing various services is reported in Table A5. 

The advent of these technological advances, along with growth in global connectivity and sharing, has given rise to companies that commercially produce the aptamers of your choice, based on a given target. SELEX commercialization, combined with academic proficiency, has given rise to numerous commercially available aptamers for a plethora of targets. All these aptamers have been target-tested and include several readily available modifications (such as 3′-his tag, Poly A, Poly T; 5′-GFP, NH, CH_3_COO) that can easily be procured by researchers or users and are readily available. This has broadened the scope for aptamers, as researchers with no prior experience in using aptamers now have access to purchase and experiment with them in developing sensors and diagnostic assays for metabolites and infectious diseases.

The process used by Lange et al. in 2012 [26], and previously described in this article, generates an innate host immunity by helping the host cell to produce its own aptamers. This model of treatment would remove the need for an external supply of aptamers and equip the cell with innate immunity in the presence of an infectious agent. Following these kinds of scientific breakthroughs in the treatment of other viral diseases like HBV, HCV and HSV is an exciting future prospect for the research community. 

### 6.2. Role of Computational Approaches in Developing Aptamers

In addition to traditional SELEX methods, a new wave of computational approaches has taken over in the discovery of high-affinity aptamers that can bind to targets of interest with high specificity. This topic has been extensively covered in review articles by Mohamad Zulkeflee et al. [209] and Chushak and Stone [210]. The groups have exhaustively reviewed the recent advances and applications of computational approaches in aptamer development. Their articles discuss pilot research focused on predicting total aptamer length and a 3D RNA aptamer structure that binds to targets of interest alongside a combination of microarray technologies, computational docking and sequence fitness modeling algorithms. Other studies have applied molecular dynamics simulations to predict whether suitable aptamers bind to proteins, peptides, small molecules, and viral antigens.

Using an extensive 3-step process, a high-affinity aptamer prediction can be made. The first step involves the selection of RNA sequences based on their secondary structure, the second step involves the generation of 3D structures, and the third step involves screening the library of RNA molecules with targets of interest using molecular docking and molecular dynamic simulation programs. While all these techniques are resource-intensive, once generated, they can be used repeatedly and with high accuracy.

### 6.3. Aptamers against the SARS-CoV-2 Pandemic

In light of COVID-19 and the global pandemic caused by the SARS-CoV-2 virus, now, more than ever, aptamer technology can direct and provide a variety of therapeutic and treatment options, especially in those cases of patients with immunocompromised or burdened systems. As of July 2021, a new study has been listed for clinical trials sponsored by Achiko AG, in collaboration with Udayana University Hospital, Indonesia for a saliva-based COVID-19 DNA aptamer test. This trial is significant as, prior to this period, aptamers for antiviral use had no clinical trials listed or published [211]. 

The study is recruiting participants for a non-randomized trial to explore diagnostic tools outside of the widely used RT-PCR method, which relies heavily on an advanced infrastructure. The proposed DNA aptamer-based test can potentially be rapid to read/diagnose and work in areas with limited infrastructure, without invasive procedures for the collection of samples (saliva examination only). The device used in the study is Aptamex, a product of Achiko’s DNA aptamer platform technology—a gold nanoparticle-based test that targets the S1 spike protein on the surface of SARS-CoV-2 and is reported to take approximately 15 min to use [211]. 

Multiple studies in the recent past have been conducted in an effort to develop diagnostic systems for the detection of SARS-CoV-2. A handful of studies also show the therapeutic benefit of using aptamers against SARS-CoV-2 and COVID-19. Yang et al. [212] have shown that their aptamer targets the receptor-binding domain of the SARS-CoV2 spike protein sub-unit 1 resulting in the neutralization of the virus. They reported six novel DNA aptamers, screened via the capillary electrophoresis (CE)-based SELEX method, designed to specifically recognize SARS-CoV-2 in human serum samples, in addition to potently inhibiting SARS-CoV-2 infection by blocking the receptor-binding domain (RBD). Conversely, Schmitz et al. [213] showed that their DNA aptamer inhibited the infection of a SARS-CoV-2 pseudovirus through a mechanism independent of the spike RBD. Their study used an automated selection process to identify an aptamer that specifically interacts with spike protein without binding to RBD.

Pramanik et al. [214] developed a theranostic system that can both detect and inactivate SARS-CoV-2. They used a gold nanostar conjugated mechanism that could rapidly detect SARS-CoV-2 antigens in 10 min through a Rhodamine 6G (Rh-6G) dye-quenching mechanism, reaching a detection limit of 130 fg/mL or approximately ~ 8 viral particles per mL. They also demonstrated that their DNA aptamer-gold nanostar conjugate can stop virus infection by blocking the ACE2 receptor-binding capability while destroying the lipid membrane of the virus. Interestingly, Ando et al. [215] propose the use of an IL-6/IL-6 receptor interaction inhibitor as a possible therapeutic against COVID-19. The IL-6 binding to the IL-6R sub-unit has been implicated in cytokine storm occurrence in the COVID-19 pathology. Their SELEX process to screen for the generation of an RNA aptamer against this interaction not only bound to IL-6R with a dissociation constant of 200 nM but also inhibited the interaction of IL-6R with IL-6. This could be an alternative route to treat the disease, rather than targeting the virus itself.

## 7. Conclusions and Future Directions

The detection and treatment of viral infections using antibodies and conventional drugs have seen success in the past. As described in this review, aptamers unequivocally provide similar if not better diagnostic and therapeutic efficiencies, with lower production costs when compared to their predecessors, and without running into issues like microorganism resistance. This can be attributed to their simple structural features, non-immunogenicity, biocompatibility, non-toxicity, and ease of production. However, their selection using the SELEX process remains a major hurdle in developing aptamers for viral infections. 

The aptamer industry has come a long way since its humble inception in the early 1990s. Today, we have access to numerous aptamers that are not only competitive with the antibody industry but are also paving the way for novel, complication-free modes of treatment. However, the research on aptamers and their prospects has only begun and will need far greater amounts of funding and focused research to unleash their true potential and results. 

From the previous sections, it is evident that some infectious agents, such as HIV and HCV, have received massive attention in the past decade, while others such as HBV and HSV have received less or no attention at all. While all these pathogens have been well studied, and their structures and lifecycles have been clearly charted out, there is disproportionate funding/research being provided/conducted for tackling these issues. Although this depends on the research focus of the funding agencies that support a research group, new reforms in research to eliminate non-lethal diseases also need to be created. In addition, further research has to be focused on translational research in the field of antiviral aptamers, for studies to reach clinical trials. This will shift the current research and healthcare industry paradigm toward using more adaptive, cost-effective and targeted technologies. 

With the advent of new companies that commercialize the aptamer generation process, there is space for fruitful collaborations between industry and academia to develop novel aptamers for the diagnosis and therapy of various diseases, such as HSV, that continue to remain evasive while carrying out tremendous damage to public health. 

## Data Availability

Not applicable.

## Appendix A

**Table A1:** List of aptamers and their properties for treatment and detection of HIV.

Reference	Detection/Therapy	Mode of Detection/Therapy	Detection Range or Binding Affinity	Target	RNA/DNA	Size	Year
[47]	Detection	-	low pM	HIV-1 RT	RNA	20 + 57 nt	2015
[46]	Detection	-	30 aM detection	-	-	-	2015
[45]	Detection	Atomic Force Microscopy	0.8 nM to 8 pM	gp120	DNA	23 kDa	2014
[43]	Detection	rGO Flourescence	-	interferon-γ	DNA	-	2014
[44]	Detection	K_d_ = 2–4 nM	28 nM	nucleocapsid protein 7 (NCp7)	RNA	20 nt SL3 Aptamer	2013
[41]	Detection	Diamond-FET-based-sensing	1 nM	HIV-1 Tat protein	RNA	19 nt Sigma Aldrich, St. Louis, MO 68178, USA	2013
[42]	Detection	Imaging	K_d_ 2.5 × 10^−8^ M	HIV-1 RT	DNA	93 nt G-Quadruplex Sangon Biotech, 698 Xiangyu Road, Songjiang District, Shanghai Zip Code: 201611 -	2011
[40]	Detection	Diamond-FET-based sensing	1 pM to 1 μg/ml	HIV 1 tat peptide	RNA	36 nt Sigma Genesis company, St. Louis, MO 68178, USA	2011
[39]	Detection and Therapy	Transfection-based assay	Prediction of best inhibitors	HIV RT	RNA	-	2014
[48]	Detection	MST Measurement	10 µM	HIV-1 integrase	DNA		2016
[49]	Detection	QIAamp kit	29 nM	gp120	RNA	29 nt	2016
[50]	Detection	qPCR		gag protein	RNA	80 nt	2017
[50]	Detection	ELISA	High affinity	RT	RNA		2017
[51]	Detection	SELEX	High affinity	RT	DNA	31 nt	2017
[52]	Detection	G-quadruplex/QTAp	High affinity	Tat protein	RNA	14 nt	2017
[53]	Therapy	AS1411	Clinical Trials	Nucleolin	RNA	-	2015
[54]	Therapy	Anti-TNPO3 siRNA	-	anti-CCR5 receptor	RNA	-	2015
[17]	Therapy	Live cell SELEX	-	anti-CCR5 receptor	RNA	-	2015
[22]	Therapy	Protease inhibition	-	aspartyl protease	RNA	-	2015
[22]	Therapy	Entry Inhibitors	-	gp120, gp41	RNA	-	2015
[19]	Therapy	Chimeras	-	gp120 or CD4	RNA	-	2015
[21]	Therapy	Stops HIV-C from causing cardiomyopathy	-	UCLA1	RNA	Modified UCLA	2014
[16]	Therapy	Aptamer shortening up to their minimal active domain	85% inhibition of HIV	5′-untranslated region of HIV-1 genome	RNA	16 nt	2014
[20]	Therapy	EC_50_ 4.9–10 µM	-	gp120	DNA	G-quadruplex-forming d(TG3AG)	2014
[18]	Therapy	RNA screening system	-	HIV RT	RNA	-	2014
[58]	Therapy	70% inhibition	K_d_ = 1.59 nM	CD40	DNA	45 nt	2014
[34]	Therapy	-	-	gp120, gp41 and HSA	DNA	G-quadruplex-forming d(TG3AG)	2014
[59]	Therapy	Functionalized Gold nano	40.2% decreased infectivity	HIV-1 RT	RNA	IDT company, 1710 Commercial Park, Coralville, Iowa 52241, USA	2013
[60]	Therapy	Hydrophobic aptamer	-	gp41 N-terminal heptad repeat (NHR)	DNA	DNA Duplex	2013
[19]	Therapy	Reduced topical dosage compard to earlier studies	-	CD4	RNA	40 nt	2013
[38]	Therapy	-	IC_50_ 10 nM	HIV-1 RT	RNA	31 nt	2013
[61]	Therapy	Binding Inhibition	-	P24 antigen	RNA	-	2013
[62]	Therapy	Rev Aptamer improvement	-	arginine-rich motif (ARM) of HIV Rev protein	RNA	30 nt	2013
[37]	Therapy	High Through-put sequencing enhances chances for better selection	-	HIV-1 RT	RNA	70 nt	2013
[63]	Therapy	IC_50_ value of 0.5 mM	K_d_ = 29–381 nM	interleukin-6	DNA	60 nt	2013
[16]	Therapy	Chemical Modification to attach siRNA	-	gp120	RNA	-	2013
[36]	Therapy	Intracellular Aptamer	5–10 fold suppression	HIV RT	RNA	-	2012
[35]	Therapy	Structural Investigation of G-Quadruplexes	-	-	DNA	G-quadruplex-forming d(TG3AG)	2012
[64]	Therapy	siRNA attached to Aptamer	-	CD4	DNA	Made from RNA Aptamer 39 nt	2012
[65]	Therapy	-	-	CCR5	DNA	23 nt	2012
[66]	Therapy	Blocking HIV translation	K_d_ = 1.28 ± 1.27 nmol/l	human cyclin T1	RNA	40 nt	2012
[67]	Therapy	-	K_d_ = 82 ± 7 nM	HIV RT	DNA	G-Quadruplex	2012
[20]	Therapy	IC_50_ 0.8 ± 0.9 nM	K_d_ = 0.15 nM	gp120	RNA	54 nt UCLA-1 first report	2012
[68]	Therapy	Stops Early intracellular events	-	Thrombin, RT	DNA	3 DNA G-Quadruplexes	2011
[69]	Therapy	Targeted Delievery of pRNA	K_d_ = 47.91 nM	gp120	RNA	81 nt Aptamer	2011
[18]	Therapy	RNA aptamer with siRNA Chimera	-	CD4	RNA	86 nt-Aptamer A1	2011
[70]	Therapy	Aptamer-siRNA Chimera	K_d_ = 47.91 nM	gp120	RNA	86 nt-Aptamer A1	2011
[71]	Therapy	HSCs are engineered to express anti-HIV molecules	K_d_ = 80 to 200 nM	*gag* polyprotein	RNA	100 nt	2011

**Table A2:** List of aptamers and their properties for treatment and detection of Hepatitis B, C and D virus subtypes.

Reference	Detection/Therapy	Mode of Detection/Therapy	Detection Range or Binding Affinity	Target	RNA/DNA	Size	Year
[90]	Detection - HBV	Chemiluminiscence	0.5ng/L	Surface Antigen	DNA	-	2015
[89]	Detection-HBV	one-step competitive binding assay	1.25 mIU mL-1	surface antigen of the hepatitis B virus	RNA	99 nt	2014
[94]	Detection-HCV	ELISA	-	glycoproteins, E1 and E2	RNA		2015
[92,93]	Detection-HCV	ELASA-First time	0.8–4 nM	HCV E2	DNA	40 nt	2013
[92]	Detection -HCV	lateral flow strip	10 pg/mL	HCV core antigen	DNA	-	2013
[95]	Detection-HCV	Malachite green flourescence	250 nM	hepatitis C helicase and replicase	RNA	-	2013
[91]	Detection-HCV	Octet interferometer	700 pg/ml	NS5B viral protein	RNA	21 nt NS5B RNA BIONEER	2011
[188]	Detection-HBV	SELEX	4 µM	HbeAg	DNA	40 nt	2016
[206]	Detection-HBV	AgNC and MoS2 nanosheets	10.7 nM	pLDH	DNA	29 nt	2017
[195]	Detection-HCV	nanoaptasensor	10 µm	HCVcoreAg	DNA		2017
[214]	Detection-HCV	ELISA	10^−10^ M	HCVcoreAg	DNA	85 nt	2018
[211]	Detection-HCV		High affinity	Ribozyme	RNA		2017
[204]	Detection-HCV	ELISA		HCV polyprotein	DNA	80 nt	2017
[78,79]	Therapy-HBV	inhibition	180 ± 82 nM	Surface protein L	DNA	-	2015
[78]	Therapy-HBV	inhibit the assembly of the nucleocapsid	-	core protein of HBV	DNA	Sangon Biotech, 698 Xiangyu Road, Songjiang District, Shanghai Zip Code: 201611	2014
[76]	Therapy-HBV	Inhibition	-	HBV P protein	RNA	29 nt	2011
[96]	Therapy-HDV	Detection of HDV Riboswitch	ON/OFF 4.7	HDV Ribozyme	RNA	8 nt	2013
[85,88]	Therapy-HCV	Chol attached Aptamer	-	nonstructural protein 5B	RNA	29 nt	2015
[88,97]	Therapy-HCV	Magnetic Separation	-	E1E2 glycoprotein	RNA	-	2015
[97]	Therapy-HCV	inhibited HCV RNA replication	-	NS2 protein	DNA	40 nt	2014
[98]	Therapy-HCV	Inhibition	-	core protein	DNA	40 nt	2014
[99]	Therapy-HCV	Inhibition	-	NS5A	DNA	40 nt	2014
[87]	Therapy-HCV	blockage of virus binding to cells	EC_50_ 62.37 nM	envelope protein (E1E2)	DNA	40 nt	2013
[84]	Therapy-HCV	competitive sequestration of the target protein	1–3 nM	HCV NS5B RNA replicas	RNA	29 nt	2013
[80,82]	Therapy-HCV	interference of HCV replication	0.4–0.5 μM	CRE-5BSL3.2 domain	RNA	30 nt	2013
[80]	Therapy-HCV	-	80% inhibition of Viral RNA	HCV-CRE194 RNA fragments	RNA	30 nt	2012

**Table A3:** List of aptamers and their properties for treatment and detection of influenza virus subtypes.

Reference	Influenza Type	Detection/Therapy	Mode of Detection/Therapy	Detection Range or Binding Affinity	Target	RNA/DNA	Size	Year
[136]		Detection	-	1 μg/mL	viral nucleoprotein and vasopressin	DNA	Nucleoprotein - 63 nt AptaRes Ag & Vasopressin-54 nt IDT company, 1710 Commercial Park, Coralville, Iowa 52241, USA	2012
[137]	influenza A/H1N1	Detection	Microfluidic SELEX	K_d_ = 55.14 ± 22.40 nM	Whole Virus	DNA	40 nt	2014
[138]	Both A and B	Detection	Gold nano attachment to virus	K_d_ = 44 ± 6 nM	hemagglutinin (HA) and neuraminidase (NA)	RNA	Various sizes	2014
[119]	H1N1	Detection	ELISA/SPR	nM range	Surface protein hemagglutinin HA1 subunit of subtype H1	DNA	G-quadruplex	2015
[139]	H3N2, H2N2, H5N1, H1N1	Detection	K_d_ Tokio 0.7 ± 0.2 and Jilin 1.2 ± 0.2 μg/mL	Tokio virus 8 ng/mL Jilin-HA 800 ng/mL	Viral SELEX	RNA	25 nt	2013
[133]	H5N1	Detection	Sandwich assay amperometric	100 fM	H5N1 specific	DNA	72 nt	2015
[131]	H5N1	Detection	Impedance	0.0128 hemagglutinin units (HAU)	H5N1 specific	DNA	-	2015
[130]	H5N1	Detection	Gold Nanogate enzymatic sensor	2–9 HAU	H5N1	DNA	-	2015
[140]	H5N1	Detection	metal-enhanced fluorescence (MEF) sensing platform	2 and 3.5 ng/mL	recombinant hemagglutinin (rHA) protein	DNA	G-quadruplex	2015
[129]	H5N1	Detection	enzymatic catalysis with electrochemical impedance	8 × 10^−4^ HAU in 200 μL	recombinant IGF-I	DNA	72-nt biotin labelled	2014
[128]	H5N1	Detection	(K_d_) of 4.65nM	12.8 HAU	hemagglutinin	DNA	N74	2013
[127]	H5N1	Detection	QCM based sensor	0.0128 to 128 HAU	H5N1 surface protein	DNA	75 nt IDT Company, 1710 Commercial Park, Coralville, Iowa 52241, USA	2013
[126]	H5N1	Detection	SPR based portable sensor	0.128 to 1.28 HAU	haemagglutinin (HA	DNA	74 nt	2012
[132]	H5N1	Detection	Electrochemical	4.3 × 10^−13^ M/L	AIV H5N1 gene sequences	DNA	23 nt Takara Biotech, 2560 Orchard Parkway, San Jose, CA 95131, USA	2011
[141]	H5N1, H1N1, and H3N2	Detection	sandwich enzyme-linked aptamer assay	K_d_ 1.53 × 10^−8^ M	HA1 protein	DNA	-	2014
[142]	Influenza A	Detection	Labelling	Visual	hemagglutinin (HA)	DNA	68 nt Sangon biotech, 698 Xiangyu Road, Songjiang District, Shanghai Zip Code: 201611	2011
[135]	-	Detection	SERS	0.1 μg/ml	Viral Nucleoprotein	DNA	22 nt AptaRes AG	2011
[103]	Influenza A	Detection	activatable silver nanoclusters beacon	High affinity	H1N1/H5N1 genes	DNA	small nt sequence	2017
[143]	H3N2	Detection	Impedimetric glycan-based biosensor	5 aM	Glycan	DNA		2016
[144]	H5N1	Detection	nanowell-based QCM aptasensor	2−4 HAU/50 μL	hemagglutin glycoprotein	DNA		2017
[145]	H9N2	Detection	RT-I-PCR	K_d_ = 40.67 nM	H9 gene	DNA	26 nt	2017
[146]	H3N2	Detection	Dual Recognition Element Lateral Flow Assay	2 × 10^−6^ virus particles	A/Panama/2007/99 (H3N2)	RNA	75 nt	2014
[122]	H5N2	Therapy	70% cell viability		hemagglutinin	RNA	40 nt	2014
[123]	H5N2	Therapy	Selection and Binding	Significant inhibition of HA	hemagglutinin	RNA	40 nt	2011
[125]	H9N2	Therapy	capillary electrophoresis SELEX		H9N2 AIV purified haemagglutinin	DNA	-	2015
[124]	H9N2	Therapy	3-fold survival	Same affinity as mouse antibody	globular region of the H9-type HA	DNA	28 nt	2011

**Table A4:** List of aptamers for various other viruses which have not been discussed in this review.

Reference	Virus	Virus Type	Detection/Therapy	Mode of Therapy/Detection	Detection Limit/Binding Affinity	Target	RNA/DNA	Size
[167]	Apple stem pitting virus		Detection	Molecularly Imprinted Polymer Gel Laser Diffraction Sensor	10 ng/mL	MT32 protein	DNA	40 nt
[168]	bovine viral diarrhea virus	BVDV type 1	Detection	Gold nano sandwich sensor	800 copies/mL	Whole Virus	DNA	30 nt
[169]	Coronavirus	SARS	Detection	Aptamer on Chip	0.1 pg/mL	nucleocapsid protein	RNA	92 nt
[170]	Norovirus	Human noroviruse	Detection	ELASA		NoV target-the P domain protein	DNA	
[171]	Norovirus	GII.2 HuNoV strain, Snow Mountain Virus	Detection		K_d_ 191 nM	Immobilized SMV	DNA	40 nt
[172]	Norovirus	Murine Norovirus	Detection	K_d_ 290 nM G-quadruplexes	20 aM to 120 aM	Viral SELEX	DNA	40 nt
[173]	Porcine reproductive and respiratory syndrome virus	VR-2332	Detection	K_d_ 2.5 × 10^5^ TCID_50_/mL	In water 0.1 × 10^0^–10^1^ TCID_50_/mL Nasal/oral fluid 4.8 × 10^0^–5.0 × 10^3^ TCID_50_/mL	Viral SELEX	DNA	40 nt
[174]	Vaccinia virus		Detection	K_d_ 26.3–40.9 nM	In water 60 PFU in 30 μL in blood 150 to 900 PFU	Viral SELEX	DNA	
[175]	vaccinia virus		Detection	Impedimetric	60 virions in a microliter	Virus Infected Cell SELEX	DNA	40 nt
[176]	vesicular stomatitis virus	Oncolytic Virus	Detection	To protect the Virus from Nuetralizing antibodies	53–86% degree of Protection	Viral SELEX	DNA	40 nt
[177]	VSV	vesicular stomatitis virus	Detection	aptamer-based affinity chromatography				switchable aptamers
[178]	Apple stem pitting virus isolates		Detection	ELONA	81% affinity	MT32/PSA-H protein	DNA	20-80 nt
[179]	Bovine Herpes Virus-1		Detection/Therapy	SELEX	3.5 nM	gD protein	DNA	38-51 nt
[180]	Dengue Virus		Detection	SELEX		5’-UTR	RNA	
[181]	Human Papilloma virus (HPV)		Detection	SELEX	10 nM	type 16 VLP	DNA	
[182]	Iridovirus		Detection	ELISA	nmol L^−1^		DNA	71 nt
[183]	Parvovirus		Detection	SELEX	467 nM		DNA	49 nt
[184]	Respiratory Syncytial Virus (RSV)		Detection	SELEX	0.5 nM	F protein	DNA	30 nt
[185]	Sindbis virus		Detection	ISH/RT-PCR	High affinity	E2 glycoprotein	RNA	
[186]	Alphavirus	TC-83 Virus	Therapy	Replicon riobozyme actuator		Vaccine	RNA	
[187]	Coronavirus	SARS	Therapy	Just binding studies	K_d_ 4–9 nM	nucleocapsid protein	DNA	45 nt
[188]	Dengue		Therapy			DENV-2 envelop protein domain III (ED3)	DNA	15 nt
[189]	Dengue	DENV-2	Therapy	Thiolated aptamers	K_d_ 154 nM	envelope protein domain III	DNA	24 nt
[190]	Ebola	VP35	Therapy	Inhibition of VP35	10–50 nM	Viral Protein 35	RNA	45 nt
[191]	foot-and-mouth disease virus		Therapy	GFP tagged aptamers	375 nM	RNA-dependent RNA polymerase	RNA	30 nt
[192]	Hemorrhagic Septicemia Virus		Therapy		Viral plaques were reduced by 50%, cells were viable and healthy	Viral SELEX	RNA	40 nt
[193]	Hirame Rhabdovirus		Therapy		15- to 63-fold reduction in plaques	Viral SELEX	RNA	40 nt
[194]	HPV	HPV-16	Therapy	Binding Inhibition		HPV-16 E7 protein	RNA	
[195]	HPV	HPV-16	Therapy		K_d_ = 0.05 pM	HPV-16 L1 VLPs	RNA	15 nt
[196]	HPV	HPV-16	Therapy	Internalization of aptamers	5 fold higher internalization	HPV-16 E6/E7 transformed tonsillar epithelial cell SELEX	RNA	30 nt
[197]	Iridovirus	Singapore grouper	Therapy	inhibition	12.09 nM	SGIV-infected GS cells	DNA	
[198]	Iridovirus	Singapore grouper	Therapy	30% less mortality of fish		Whole Virus	DNA	50 nt
[199]	Norovirus		Therapy			capsid protein VP1	DNA	49 nt
[200]	rabies		Therapy	25–33% survival	K_d_ 307 nM	RABV glycoprotein	DNA	45 nt
[201]	rabies		Therapy	30–50% survival		Cell SELEX	DNA	
[202]	rabies		Therapy	4 nmol PEG-FO24 protected 87.5%		RABV-infected BHK-21	DNA	45 nt
[203]	rabies		Therapy		K_d_ 28–39 nM	Virus infected Cell SELEX	DNA	45 nt
[204]	Rift Valley fever virus		Therapy			nucleocapsid protein	RNA	30 nt
[205]	xenotropic murine leukemia virus-related virus (XMRV)	Different from HIV-1	Therapy	IC_50_ 2 nM		XMRV RT	RNA	three 12 nt sequences

**Table A5:** List of companies offering various aptamer-based services. All the data presented here has been collected from the respective company websites.

Company	Custom Aptamers	SELEX Kits	Catalogue Aptamers	Random DNA Library	Clinical Trials	Country
IDT, 1710 Commercial Park, Coralville, Iowa 52241, USA	No	No	No	Yes	No	USA
AMSBio, 1035 Cambridge St Ste 16B, Cambridge, Massachusetts 02141-1154, USA	Yes	Yes	No	Yes	No	Spain
Gene Link, Inc. 8 Westchester Plaza, Suite 130, Elmsford, NY 10523, USA	Yes	No	No	Yes	No	USA
Roboklon, Robert-Rössle-Str. 10, 13125 Berlin, Germany	Yes	Yes	No	Yes	No	Germany
Ambiotech, 225 Broadway, #1903, New York, NY 10007, USA	Yes	Yes	No	Yes	No	USA
Jena Bioscience, Löbstedter Str. 71, 07749 Jena, Deutschland	Yes	No	No	Yes	No	Germany
TriLink, 10770 Wateridge Cir Ste 200, San Diego, CA 92121, USA	Yes	Yes	No	Yes	No	USA
IBA Lifesciences, Rudolf-Wissell-Str. 28, 37079 Göttingen, Germany	Yes	No	No	Yes	No	Germany
Neoventures, 516 Colborne St, London ON	Yes	No	No	Yes	No	Canada
OTC Biotech, San Antonio, Texas, USA	Yes	No	Yes	Yes	No	USA
Aptagen, Aptagen, LLC, 250 North Main Street, Jacobus, PA 17407, USA	Yes	No	Yes	Yes	No	USA
BasePair Technologies, 415 Madison Ave fl 4, New York, NY 10017, USA	Yes	No	Yes	Yes	No	USA
AptiSci, 301 Ho, 3 Dong, Pangyo Seven Venture Valley, Republic of Korea	Yes	No	Yes	Yes	No	South Korea
CamBio, 1 The Irwin Centre, Scotland Road, Dry Drayton, Cambridge, UK	No	No	From BasePair Technologies	Yes	No	UK
SomaLogic, 2945 Wilderness Pl. Boulder, CO 80301, USA	No	No	No	No	Yes	USA
Noxxon Pharma AG, Max-Dohrn-Strasse 8-10, 10589 Berlin, Germany	No	No	No	No	Yes	Germany
Gilead Sciences, Inc. 333 Lakeside Drive, Foster City, CA 94404, USA	No	No	No	No	Yes	USA
Sangon Biotech, 698 Xiangyu Road, Songjiang District, Shanghai Zip Code: 201611	No	No	No	Yes	No	China
Takara Bio Inc, 2560 Orchard Parkway, San Jose, CA 95131, USA	No	No	No	Yes	No	China
AptaRes AG, Am Scheunenviertel 1, 15749 Mittenwalde, Germany	No	No	No	Yes	No	Germany
Bioneer, Inc. 155 Filbert St, Ste. 216, Oakland, CA 94607, USA	No	No	No	Yes	No	USA
Sigma Aldrich, St. Louis, MO 68178, USA	No	No	No	Yes	No	USA
